# Prevalence of hypothermia and critical hand temperatures during military cold water immersion training

**DOI:** 10.1080/22423982.2023.2236777

**Published:** 2023-07-20

**Authors:** Douglas M. Jones, Rebecca S. Weller, Rebecca J. McClintock, Nicholas Roberts, Weimin Zheng, Timothy L. Dunn

**Affiliations:** a Warfighter Performance, Naval Health Research Center, San Diego, CA, USA; b Mountain Medicine, Marine Corps Mountain Warfare Training Center, Bridgeport, CA, USA

**Keywords:** Cold-injury, warfighter, cold-weather medicine, core temperature, hand function

## Abstract

Cold-weather military operations can quickly undermine warfighter readiness and performance. Specifically, accidental cold-water immersion (CWI) contributes to rapid body heat loss and impaired motor function. This study evaluated the prevalence of hypothermia and critical hand temperatures during CWI. One-hundred seventeen (*N* = 117) military personnel (mean ± SD age: 27 ± 6 yr, height: 176 ± 8 cm, weight: 81.5 ± 11.6 kg) completed CWI and rewarming during cold-weather training, which included a 10-min outdoor CWI (1.3 ± 1.4°C) combined with cold air (−4.2 ± 8.5°C) exposure. Following CWI, students removed wet clothing, donned dry clothing, and entered sleeping systems. Core (T_c_) and hand (T_hand_) temperatures were recorded continuously during the training exercise. T_c_ for 96 students (mean ± SD lowest T_c_ = 35.6 ± 0.9°C) revealed that 24 students (25%) experienced T_c_ below 35.0°C. All of 110 students (100%) experienced T_hand_ below 15.0°C, with 71 students (65%) experiencing T_hand_ at or below 8.0°C. Loss of hand function and hypothermia should be anticipated in warfighters who experience CWI in field settings. Given the high prevalence of low T_hand_, focus should be directed on quickly rewarming hands to recover function.

## Introduction

Warfighters perform operational duties in harsh environments that include hazards such as cold temperatures, water immersion, and/or prolonged exposure to a combination of environmental and operational stressors [1,2]. For example, infantry may need to cross a cold river or wade through chest-high marshlands to reach a critical extraction point, or sailors may incur elevated risk of cold air exposure and water immersion when sailing through hostile waters in cold regions of the world [3,4]. Exposure to cold stress can undermine performance through dexterity loss, mild hypothermia, shivering, pain, discomfort, and cognitive distraction, leading to attenuated vigilance and decision-making errors [5–8]. In more severe exposures, cold stress may precipitate freezing and non-freezing injuries requiring medical evacuation, such as frostbite, immersion (trench) foot, or moderate to severe hypothermia [9,10]. Although several actions can be taken to reduce the likelihood or intensity of cold exposure in the field (e.g. detailed mission planning, proper route selection, adequate thermal protection, frequent monitoring of weather forecasts), cold exposure may still be unexpected [2,11]. It is therefore prudent to understand the probable incidence of cold-weather casualties when mission plans change, thermal protection is damaged or lost, or weather and situations abruptly deteriorate.

Cold-weather injury data from the Defense Health Agency’s most recent Medical Surveillance Monthly Report indicate that approximately 500 cold-weather injuries are reported each year across the U.S. military branches. Reported cold-weather injuries include frostbite, immersion injury, and hypothermia (55%, 30%, and 15% of all cold injuries, respectively [12]. This information is critical to recognising the impact that cold exposure has on military operations and is of great importance to understanding casualty rates in cold environments. However, these data fail to capture the warfighters who experience cold stress, acutely deteriorate (physiologically and/or cognitively), encounter performance impairments, and then recover in the field. This scenario is difficult to detect and collect information on in field settings. However, it is important to give attention to this scenario because it is a precursor to cold injuries and elevates risk for operational mishaps resulting from impaired performance [13,14]. Understanding performance decrements during cold exposure in field settings is aided by physiological monitoring. Specifically, measurements of hand (T_hand_) and core (T_c_) temperatures obtained during cold exposure provide valuable insight into the magnitude and rate of warfighter deterioration prior to the onset of any cold injury. For example, critical T_hand_ of 15.0°C and 8.0°C are defining points for dexterity loss and complete nerve block (full loss of sensation and function), respectively, and T_c_ monitoring can be used to gauge cold stress severity through monitoring rates of body heat loss [6,15–17]. Field physiological monitoring can therefore be used to identify anticipated outcomes in specific scenarios when large groups are involved. Anticipated outcomes supported by high-quality data promote confidence among military leadership when they must make critical operational decisions in the field, such as determining how much recovery time will be needed following cold stress or understanding the degree of performance impairment associated with a cold river crossing. For example, factoring an additional 30 min to allow for rewarming/recovery following a cold river crossing may be all that is needed to execute an on-time arrival at an extraction point. Thus, using high-quality data captured from physiological monitoring in field settings can greatly improve field decision-making and operational readiness.

The purpose of this study was to evaluate the prevalence of hypothermia and critical T_hand_ during military cold-water immersion (CWI) training in a field setting. Based on previous observations of warfighters performing CWI training in field settings, we hypothesised that (1) mild hypothermia would be detected in approximately one third of warfighters, and (2) critical T_hand_ of 15.0°C and 8.0°C would be experienced by most warfighters. Understanding the prevalence of warfighter deterioration in cold environments will allow military leadership and medical providers to anticipate warfighter readiness and provide preventive guidance and care prior to the onset of a cold injury.

## Methods

### Participants

One hundred seventeen (*N* = 117; male = 108, female = 9) active-duty military personnel (mean ± *SD* age: 27 ± 6 years, height: 176 ± 8 cm, weight: 81.5 ± 11.6 kg) participated in field CWI as part of cold-weather training course requirements. Following dissemination of study details and procedures, participants provided voluntary informed consent to have their T_c_ and T_hand_ monitored and evaluated during their involvement in field CWI training exercises. The study was approved by the Institutional Review Board at the Naval Health Research Center (Protocol No. NHRC.2019.0007).

### Cold water immersion – field training exercise

Per course procedures, students participating in CWI field training exercises were instructed to enter a cold-water pond in standard battle dress uniform, briefly submerse their heads, and remain immersed in cold water to the level of the neck for 10 min. Following immersion, students were instructed to exit the water and perform cognitive tasks for 10 min (still in wet clothing) prior to changing into dry clothing. Once in dry clothing, students entered sleeping bags and performed passive rewarming exercises for up to 60 min. All training exercises occurred outdoors, from 0500 to 1000, and during winter months (January – March) in prevailing environmental conditions (see Results section for air and water temperature values).

### Experimental protocol

Data were collected across seven separate trainings occurring each winter over 4 years and the protocol for each training was identical. All participants were provided ingestible T_c_ capsules (ingested 7 hr prior to immersion) and data recorders to capture minute-by-minute T_c_ data during the CWI training (VitalSense®, Phillips Respironics, Bend, OR, USA). Additionally, VitalSense dermal temperature sensors were adhesively affixed to each participant’s right hand (posterior) for continuous monitoring and collection of T_hand_ during CWI training. Research staff measured air and water temperature multiple times during each CWI training using handheld instruments (air: Kestrel 5500®, Weather Metre, Boothwyn, PA, USA; water: Hanna® Instruments HI93532 Dual Input K-Type Thermocouple Thermometer, Smithfield, RI, USA). T_c_ and T_hand_ data were analysed to determine the number of participants who achieved T_c_ <35.0°C and the number of participants who achieved T_hand_ ≤15.0°C and ≤ 8.0°C. These values were chosen due to their clinical significance (e.g. hypothermia) or strong associations with changes in physiology and/or function (e.g. critical T_hand_). As previously mentioned, dexterity loss is commonly observed when T_hand_ reaches 15.0°C, and complete nerve block (total loss of sensation and function) is associated with T_hand_ ≤8.0°C [6,16].

## Statistical analysis

Frequency analysis was used to identify the number of participants who achieved T_c_ <35.0°C and T_hand_ ≤15.0°C and ≤ 8.0°C. Data are presented as participant counts and as percentages of total participants. Onset time to 35.0°C (T_c_) and 15.0°C and 8.0°C (T_hand_) were also evaluated to determine cooling rates and the expected times that warfighters and leadership can expect for the onset of hypothermia, dexterity loss, and tactile sensation loss. Additionally, linear regression analysis was performed to evaluate the relationship between T_c_ and T_hand_. Alpha level was set at *p* < .05 to determine correlation significance.

## Results

**Figure 1.**
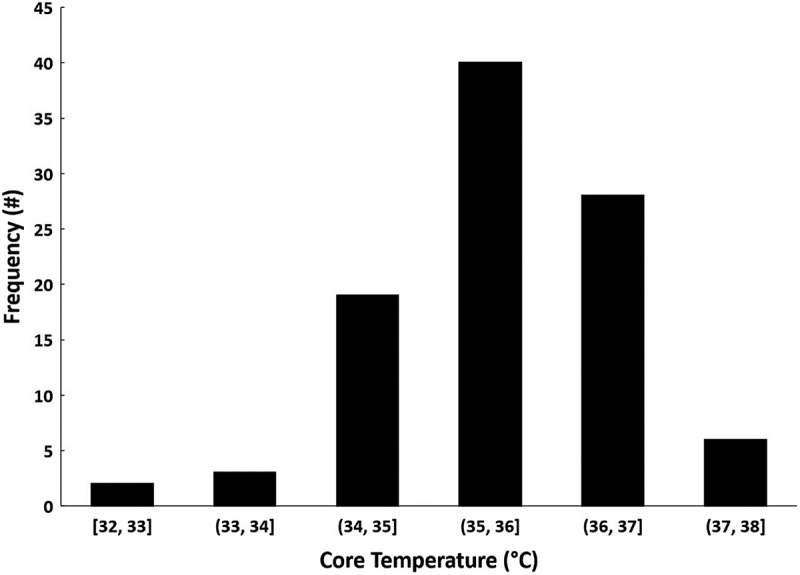
Frequency counts for 96 participants, representing their lowest T_c_ recorded during CWI training exercises. Value ranges on the *x*-axis indicate the temperature ranges in which participants’ lowest T_c_ were recorded (e.g. a T_c_ of 33.6°C would be placed in the “33, 34” range).

**Figure 2.**
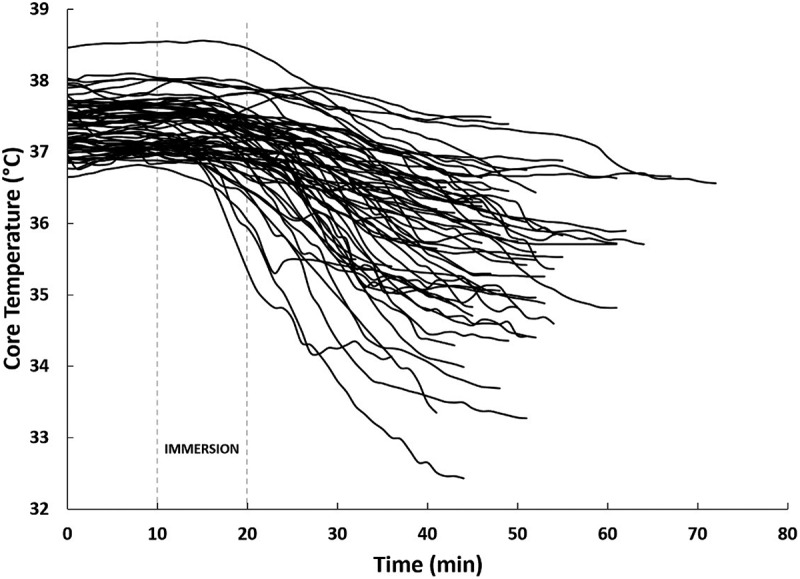
Continuous T_c_ data (recorded and displayed each minute) from the start of training exercises (i.e. before immersion) to their lowest T_c_ recorded. The data displayed are only a subset (*n* = 66) of all participants and demonstrate the significant variability in T_c_ response to 10-min CWI. Note that Figure 3 calculates hypothermia onset time from the start of immersion (which is displayed as Minute 10 in this figure).

**Figure 3.**
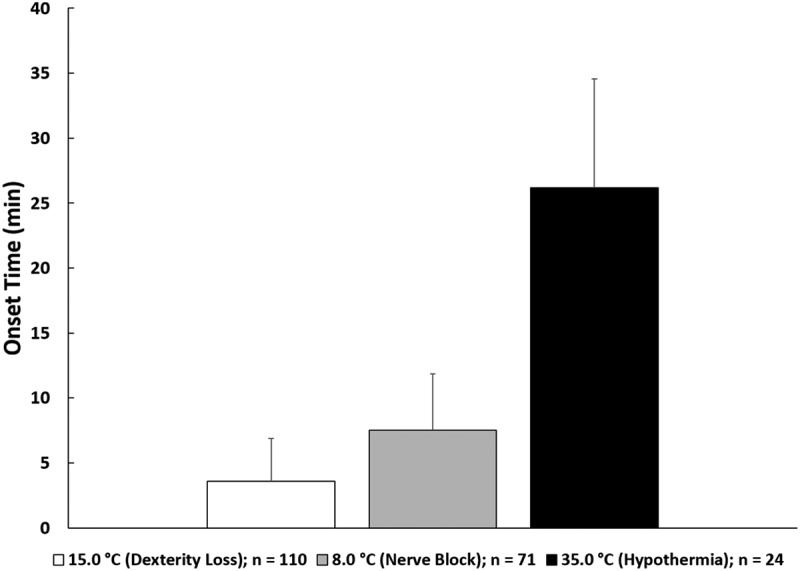
Onset times for critical T_hand_ (15.0°C and 8.0°C) and hypothermia (35.0°C T_c_), which were calculated from the start of immersion (i.e. Minute 0) to the point at which the lowest temperature was recorded.

Mean ± *SD* values for air and water temperature across all seven trainings were − 4.2 ± 8.5°C and 1.3 ± 1.4°C, respectively. Of the 117 participants who performed CWI during the seven trainings, T_c_ and T_hand_ were obtained on 96 and 110 students, respectively (some data loss occurred due to students inadvertently separating from their data recorders during training or T_hand_ sensors separating from the participant). Evaluation of T_c_ for 96 participants (mean ± *SD* lowest T_c_ = 35.6 ± 0.9°C) revealed that 24 participants (25%) experienced T_c_ below 35.0°C (Figure 1). Further analysis of T_c_ data suggests that in the 10 min prior to immersion, T_c_ was relatively homogenous among the 96 participants, given the small standard deviation (mean ± *SD*: 37.4 ± 0.3°C). T_c_ homogeneity was also observed during immersion (mean ± *SD*: 37.2 ± 0.4°C). However, in the 20 min after exiting the water, the variability of T_c_ increased significantly (mean ± *SD*: 36.3 ± 0.9°C) (Figure 2). For the 24 participants who experienced T_c_ <35.0°C (i.e. hypothermia), the onset time for reaching 35.0°C, from the first minute of immersion, was 26 ± 8 min (range: 11–45 min) (Figure 3).

Note that functional responses for T_hand_ (i.e. dexterity loss, nerve block) in brackets were not measured. Here this represents the anticipated functional effects of reaching these specific T_hand_ [6].

**Figure 4.**
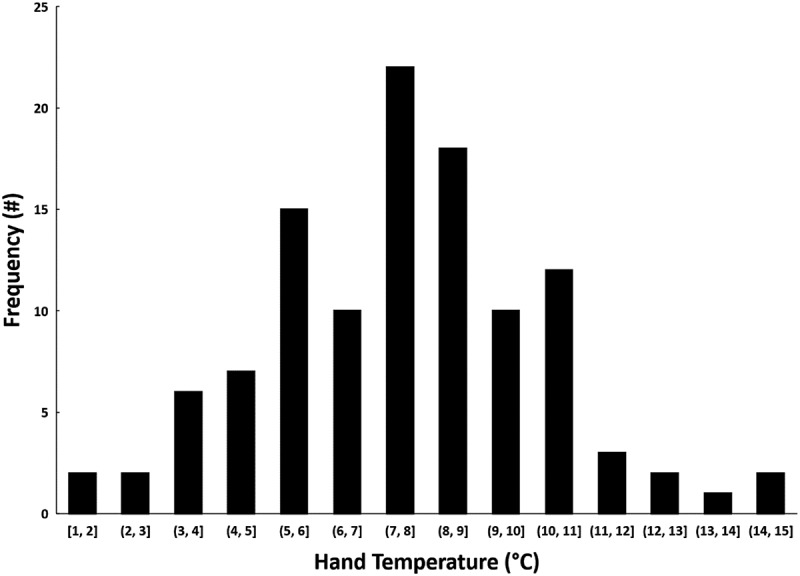
Frequency counts for 110 participants, representing their lowest T_hand_ recorded during CWI training exercises. Value ranges on the *x*-axis indicate the temperature ranges in which participants’ lowest T_hand_ were recorded (e.g. a T_hand_ of 7.2°C would be placed in the “7, 8” range).

For T_hand_ (mean ± *SD* lowest T_hand_ = 7.6 ± 2.5°C), all 110 participants (100%) experienced T_hand_ below 15.0°C, with 71 participants (65%) experiencing T_hand_ at or below 8.0°C (Figure 4). Linear regression suggests no relationship between T_c_ and T_hand_ throughout the CWI training (*r* = 0.09, *p* = .390). T_hand_ was also analysed to determine onset times to 15.0°C and 8.0°C from the first minute of immersion, which revealed an onset time of 4 ± 3 min to 15.0°C and, for the 71 participants who experienced T_hand_ at or below 8.0°C, the onset time to 8.0°C was 8 ± 4 min (Figure 3).

## Discussion

The purpose of this study was to evaluate the incidence of hypothermia and critical T_hand_ during a military CWI field training exercise. The primary findings from this work suggest that (1) mild hypothermia should be expected in approximately 25% of warfighters who enter cold water (<5°C) for up to 10 min, with an anticipated onset time of 30 min, although onset could appear sooner or later, and (2) dexterity loss will likely occur within minutes of CWI and should be expected to impact nearly all warfighters. Specific attention should be given to the environmental temperatures and exposure durations that developed these findings, as adjustments to temperature or exposure duration will alter these outcomes. T_c_ responses to cold stress are often highly variable given the multiple combinations of individual and environmental factors that influence body heat loss [18]. For example, immersion in cold water of 5–10°C produces T_c_ cooling rates of 0.06°C/min (hypothermia onset time = 30–35 min, assuming a starting T_c_ of 37°C), whereas immersion in water of 15–20°C (still defined as cold water), only produces cooling rates of 0.02°C/min (hypothermia onset time = 100 min, assuming starting T_c_ of 37°C) [2]. As such, hypothermia onset would be expected to take much longer to develop during less intense CWI. In the current study, we measured an average water temperature of 1.3°C and observed an average hypothermia onset time of 26 min (calculated from the start of immersion, Minute 0), which is in alignment with cooling rates presented by Wittmers and Savage [2]. The water temperature remained consistent across the seven training sessions, yet we still observed large variations in T_c_ responses among warfighters following immersion.

These variations were most likely related to varying intensities of cold air exposure (upon exiting the water) that were experienced during the different training events and individual differences in body composition, as subcutaneous fat is shown to influence cooling rates during cold exposure [19]. Such variations should be expected in warfighters who experience CWI during field operations.

Stages of hypothermia are frequently defined in medical literature using T_c_ ranges, such as mild (32–35°C), moderate (28–32°C) and severe (≤28°C). Each stage is associated with changes in physiology, function, and behaviour [20]. In the current study, the lowest T_c_ recorded was 32.4°C, indicating presence of mild hypothermia and progression towards moderate hypothermia. For the 24 participants who did experience mild hypothermia during the course of the training exercises, all recovered to normal body temperatures in the hours following immersion (most recovered completely within 60–70 min following immersion by performing passive rewarming, conducted as part of the training). Additionally, the most frequent T_c_ range we observed was 35–36°C, indicating that most did not experience any stage of hypothermia (per the clinical definition of hypothermia). These findings should be considered by military leadership, because (1) despite mild hypothermia observed in 24 warfighters, all recovered with no issues; and (2) the majority of warfighters will experience some degree of body heat loss but not progress to hypothermia. As stated previously, these findings should be interpreted within the contexts of the environmental conditions and exposure times associated with this study.

Although hypothermia is a significant medical concern that must be anticipated during cold-weather operations, our data suggest that low T_hand_ is much more prevalent and, therefore, could have an extensive impact on operational readiness. Consequences of low T_hand_ and dexterity loss include an inability to operate equipment and weapons, provide medical care, execute field survival skills, or maintain cognitive adaptability [21,22]. Cooper et al. [23] demonstrated that time to complete an intravenous needle insertion prior to CWI took 70 s, whereas following immersion the same procedure took 168 s (140% increase in time). Had this been a live patient in a real medical emergency requiring intravenous therapy, the outcome could have resulted in prolonged treatment time. The underlying physiological causes of dexterity loss appear to be associated with multi-factorial decrements, as opposed to a single factor responsible for dexterity loss. These reactions to cold include increased viscosity of synovial (joint) fluid, dampening of tactile receptor sensitivity, attenuated nerve conduction velocity, and decreased muscular contraction force [6]. As previously mentioned, critical T_hand_ of 15.0°C and 8.0°C are strongly associated with physiological changes and pain, resulting in reduced hand function. Our observations indicate that all participants experienced T_hand_ of 15.0°C within minutes of CWI. As such, initial decrements in dexterity should be expected under these conditions in all exposed persons. We also observed 65% of participants reach 8.0°C T_hand_, and it is likely that these participants experienced significant loss of hand function. Given the high prevalence of reaching critical T_hand_ under these conditions, military leaders operating in similar conditions should prepare by providing adequate time to rewarm and appropriate thermal protection, and by increasing warfighter knowledge about the expected outcomes of cold stress.

A major limitation of the current study is that we did not measure hand function in the cold; therefore, we can only presume that hand function was impaired upon reaching these critical T_hand_. Evidence from other studies (noted previously) do indicate a strong relationship between these temperatures and hand function. Although no objective measurements of hand function were obtained, we did observe most participants struggle with changing clothes (e.g. unbuttoning shirts, using zippers) following CWI and they often required assistance from others while changing into dry clothing. Additionally, given the high variability of human responses to cold and the multiple factors that influence such responses, these findings can only be applied to similar conditions.

Warfighters operating in cold environments should anticipate a high prevalence of cold hands and associated impacts on dexterity and function. Hypothermia should also be expected, but with much less prevalence. By anticipating and understanding the influence that cold has on human performance and function, military leaders can take appropriate actions to minimise risks to operational readiness. Future research efforts should further refine these findings by including objective measurements of military-relevant tasks, which would not only supplement the findings here, but also provide valuable knowledge on expected performance decrements in operational settings.

## Disclaimer

I am a military service member or federal/contracted employee of the U.S. Government. This work was prepared as part of my official duties. Title 17, U.S.C. §105 provides that copyright protection under this title is not available for any work of the U.S. Government. Title 17, U.S.C. §101 defines a U.S. Government work as work prepared by a military service member or employee of the U.S. Government as part of that person’s official duties. Report No. 22–69 was supported by the Defense Health Agency (restoral funding) under work unit no. N1804. The views expressed in this work are those of the authors and do not necessarily reflect the official policy or position of the Department of the Navy, Department of Defense, nor the U.S. Government. The study protocol was approved by the Naval Health Research Center Institutional Review Board in compliance with all applicable Federal regulations governing the protection of human subjects. Research data were derived from an approved Naval Health Research Center Institutional Review Board protocol, number NHRC.019.0007.
